# Dataset of the nutritional composition of follow-on infant formulas commercialized worldwide in 2021

**DOI:** 10.1016/j.dib.2023.109649

**Published:** 2023-10-05

**Authors:** Mathilde Cancalon, Youna M. Hemery, Nathalie Barouh, Rallou Thomopoulos, Bruno Baréa, Erwann Durand, Pierre Villeneuve, Claire Bourlieu Lacanal

**Affiliations:** a CIRAD, UMR Qualisud, F34398 Montpellier France; b Qualisud, Univ Montpellier, Avignon Université, CIRAD, Institut Agro, IRD, Université de La Réunion, Montpellier, France; c UMR IATE, UM Montpellier, INRAE, Institut Agro, F34060 Montpellier France; d IRD, UMR Qualisud, F34398 Montpellier France

**Keywords:** Infant follow-on formulas, Nutrition, Ingredients list, Nutritional values, Lipids, Emulsifiers, Vitamins

## Abstract

The main objective of infant follow-on formulas, consumed from the age of 6 to 12 months, is to mimic the composition of breast milk in order to meet the nutritional needs of infant. In this context, their composition is governed in Europe by a strict regulation that has evolved in 2020 to force manufacturers to improve the nutritional profile of the formulas. The objective of this dataset was to collect the ingredient lists and nutritional values of infant follow-on formulas present on the world market with a focus on the lipid fraction. The data collection was carried out from December 2020 to April 2021 directly on the product packaging or on the websites of the different brands. Only “classic” infant follow-on formulas that are widely consumed were listed. Thus, the ingredient lists and nutritional values of 91 infant formulas were collected. The nutritional values are systematically presented for 100 g of powder, for 100 Kcal and for 100 mL of formula. The sources of fats, emulsifiers and vitamins A and E were also extracted from the ingredient lists. This dataset can be used as a tool for the formulation of infant follow-on formulas or to situate the positioning of products in relation to the market.

Specifications Table

**Table:** 

Subject	Health and medical sciences: Nutrition
Specific subject area	Composition and nutritional values of infant follow-on formulas
Data format	Raw and, Filtered
Type of data	Table
Data collection	The data were collected from brand websites or directly from product labels according to three selection criteria: - The products had to be follow-on infant formulas, i.e. consumed between the ages of 6 and 12 months - They had to be so-called “classic” formulas (not hypoallergenic, anti-reflux …) - The formulas had to be widely consumed to cover at least 60 % of the market share All data were anonymized via a coding of the products from IF1 to IF91 and a standardization of the ingredients lists (decreasing weight order not necessarily respected).
Data source location	Global
Data accessibility	Repository name: Dataverse INRAE Data identification number: 10.57745/CLER60 Direct URL to data: https://doi.org/10.57745/CLER60
Related research article	M. Cancalon, Y. M. Hemery, N. Barouh, B. Baréa, C. Berton-Carabin, L. Birault, E. Durand, P. Villeneuve and C. Bourlieu- Lacanal, Comparison of the effect of various sources of saturated fatty acids on infant follow-on formulas oxidative stability and nutritional profile. Food Chem. [1]

## 1 Value of the Data

• These data are useful to companies or researchers in the field of infant nutrition
• The dataset is a tool to help in the formulation of infant formulas for companies or representative model systems for laboratory research
• Companies or intervention programs aimed at improving infant formulas composition can use our data as a benchmark
• Our data are useful to companies to position their product in relation to the market and regulations
• The dataset can be used to identify the most used raw materials with a focus on the lipidic fraction (oils mixtures, sources of vitamins A and E fortification, type of emulsifiers)
• These data constitute a support for the estimation of the nutritional intake and the contribution of each ingredient. These data are complementary to the CIQUAL, USDA or FCEN databases

## 2 Data Description

This dataset was generated to provide an overview of the ingredient lists and nutritional values of 91 infant follow-on formulas covering more than 60 % of the market share by volume. The nutritional characteristics of infant follow-on formulas have been compared with European and international regulations. This overview was also intended to identify the sources of fat, emulsifiers and fat-soluble vitamin fortification (A and E) most commonly used in this type of product (Table 1).

**Table 1:** Nutritional characteristics of infant follow-on formulas from the dataset and European and international regulations.

(/100 mL)	Infant follow-on formulas Mean ± SD	EU regulation *CE 2016/127*		*Codex Alimentarius* *CX 156/1987*	
		Min	Max	Min	Max
**Energy (kcal)**	67,2 ± 1,3	60	70	60	85
**Lipids (g)**	3,3 ± 0,2	2,64	4,2	1,8	5,1
SFA (g)	1,1 ± 0,4	–	–	–	–
MUFA (g)	1,5 ± 0,4	–	–	–	–
PUFA (g)	0,6 ± 0,1	–	–	–	–
LA (mg)	502,2 ± 0,1	300	840	180	–
ALA (mg)	53,7 ± 12,6	30	70	–	–
ARA (mg)	9,4 ± 5,6	–	42	–	–
DHA (mg)	14,8 ± 4,0	12	35	–	–
**Carbohydrates (g)**	7,8 ± 0,4	5,4	9,8	–	–
**Proteins (g)**	1,4 ± 0,2	1,08	1,75	1,8	4,7
Casein (g)	0,8 ± 0,3	–	–	–	–
**Vitamin A (µg)**	61,5 ± 6,4	42	79,8	45	191,3
**Vitamin D (µg)**	1,4 ± 0,3	1,2	2,1	0,6	2,55
**Vitamin E (mg)**	1,2 ± 0,4	0,36	3,5	0,28	–
**Vitamin C (mg)**	9,8 ± 2,3	2,4	21	4,8	–
**Iron (mg)**	0,98 ± 0,16	0,36	1,4	0,6	1,7
**Copper (mg)**	0,052 ± 0,06	0,036	0,070	–	–

SFA: Saturated fatty acid; MUFA: Monounsaturated fatty acid; PUFA: Polyunsaturated fatty acid; LA: Linoleic acid; ALA: α-linolenic acid; ARA: Arachidonic acid; DHA: Docosahexaenoic acid.

The dataset is provided as a text file (.txt) and three Excel Spreadsheet (.xlsx) files, each including one sheet:

***Summary.txt*** provides information about the aim and the method used to generate this dataset in three points. The “aim of the dataset” section describes the objective, the “Selection criteria” section lists the criteria for data inclusion and the “Data collection method” section defines the data collection method used. This file also gives additional information such as the abbreviations list and the specificities on the list of ingredients (decreasing order of weight of ingredients not necessarily respected) and the sources of vitamins A and E (addition of an asterisk when the source is not specified).

**Fig. 1:**
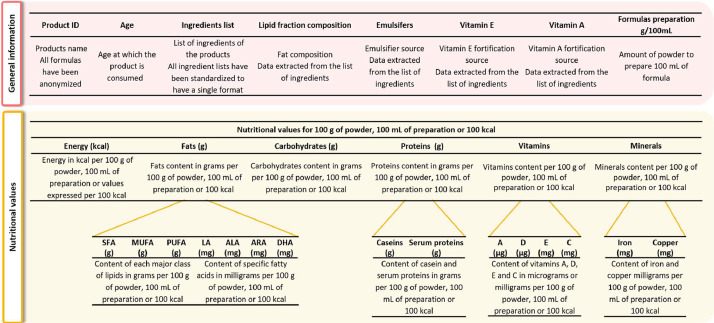
Organization and information provided in the “Nutritional values.xlsx” file.

***Nutritionalvalues.xlsx*** provides information about the age group in which the product is consumed, the list of ingredients, the composition of the oil mixture, the source of emulsifier and vitamin A and E fortification, the preparation conditions per 100 mL and the nutritional values per 100 g of powder, 100 mL of preparation or 100 kcal. Thus energy, fats content with details on the content of saturated fatty acids (SFA), monounsaturated (MUFA) and polyunsaturated fatty acids (PUFA) and specific fatty acids (linoleic (LA), α-linolenic (ALA), docosahexaenoic (DHA) and arachidonic acids (ARA)) are listed. The carbohydrates and proteins contents (with casein and serum protein contents) are also provided. Finally, the contents of vitamins A, E, C, D and iron and copper are presented. When the values were not available the boxes are left empty. This file is organized as described in Fig. 1.

***Overview.xlsx*** provides information about the number of values available on the product labels or on the website, the average (with standard deviations), maximum and minimum nutritional values for 100 g of powder, 100 ml of preparation or 100 kcal.

***Exemples of regulation.xlsx*** provides information concerning the range of contents in certain macro and micronutrients governed by the international regulation (*Codex Alimentarius* STAN 156/1987) and the European regulation (UE regulation 2016/127).

## 3 Experimental Design, Materials and Methods

**Fig. 2:**
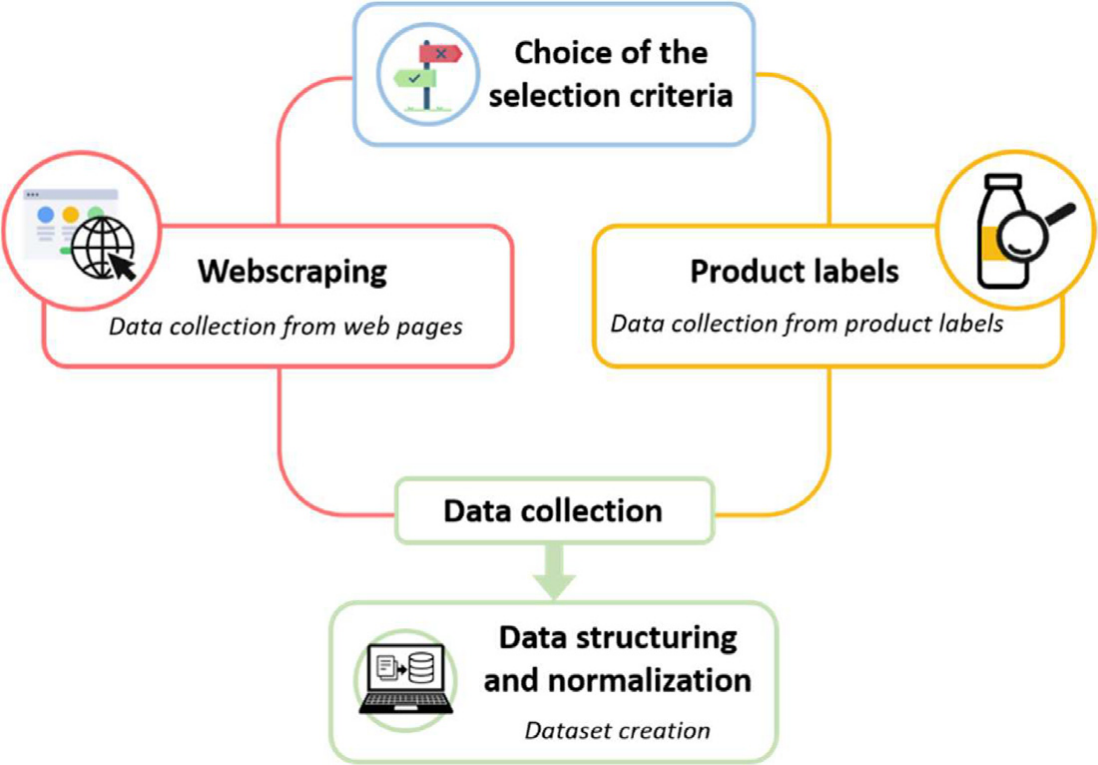
Collection data method.

The data were collected according to a three-phase method as shown in Fig. 2. In a first step, selection criteria were established. Thus, the products included in the dataset had to be infant formulas of second age also named follow-on formulas. They had to be classic formulas (no anti-reflux or hypoallergenic formulas…) and widely consumed. The data were then collected in two ways. Either via a webscraping method (information collected on websites either of brands or merchants trading products) or directly on the labels of the products. Once the data collected, it was anonymized by attaching a product code to each formulas and then structured to form the dataset. The lists of ingredients were standardized in order to preserve the anonymization and to present the data systematically in the same format. In each list of ingredients, data were extracted when they were indicated, namely the sources of fat, emulsifier and vitamin A and E used.

## Limitations

The data included in the dataset is subject to change, particularly in case of regulatory changes, and therefore needs to be regularly updated.

## Ethics Statement

The authors have read and follow the ethical requirements for publication in Data in Brief and confirming that the current work does not involve human subjects, animal experiments, or any data collected from social media platforms.

## CRediT authorship contribution statement

**Mathilde Cancalon:** Conceptualization, Methodology, Investigation, Writing – review & editing. **Youna M. Hemery:** Conceptualization, Methodology, Writing – review & editing, Validation. **Nathalie Barouh:** Conceptualization, Methodology, Writing – review & editing, Validation. **Rallou Thomopoulos:** Resources. **Bruno Baréa:** Resources. **Erwann Durand:** Writing – review & editing, Validation. **Pierre Villeneuve:** Supervision, Writing – review & editing, Validation. **Claire Bourlieu Lacanal:** Supervision, Conceptualization, Methodology, Writing – review & editing, Validation.

## Data Availability

Panorama of the nutritional composition of follow-on infant formula commercialized worlwide in 2021 (Reference data) (Dataverse)

## References

[1] Cancalon M. (2023). Comparison of the effect of various sources of saturated fatty acids on infant follow-on formulas oxidative stability and nutritional profile. Food Chem..

